# A case control study examining the feasibility of using eye tracking perimetry to differentiate patients with glaucoma from healthy controls

**DOI:** 10.1038/s41598-020-80401-2

**Published:** 2021-01-12

**Authors:** Andrew J. Tatham, Ian C. Murray, Alice D. McTrusty, Lorraine A. Cameron, Antonios Perperidis, Harry M. Brash, Brian W. Fleck, Robert A. Minns

**Affiliations:** 1 University of Edinburgh, Edinburgh, UK; 2 Princess Alexandra Eye Pavilion, 45 Chalmers Street, Edinburgh, EH3 9HA UK; 3 Glasgow Caledonian University, Glasgow, UK; 4 Heriot Watt University, Edinburgh, UK; 5 Royal Hospital for Sick Children, Edinburgh, UK

**Keywords:** Diagnosis, Optic nerve diseases

## Abstract

To explore the feasibility of using Saccadic Vector Optokinetic Perimetry (SVOP) to differentiate glaucomatous and healthy eyes. A prospective case–control study was performed using a convenience sample recruited from a single university glaucoma clinic and a group of healthy controls. SVOP and standard automated perimetry (SAP) was performed with testing order randomised. The reference standard was a diagnosis of glaucoma based a comprehensive ophthalmic examination and abnormality on standard automated perimetry (SAP). The index test was SVOP. 31 patients with glaucoma and 24 healthy subjects were included. Mean SAP mean deviation (MD) in those with glaucoma was − 8.7 ± 7.4 dB, with mean SAP and SVOP sensitivities of 23.3 ± 0.9 dB and 22.1 ± 4.3 dB respectively. Participants with glaucoma were significantly older. On average, SAP sensitivity was 1.2 ± 1.4 dB higher than SVOP (95% limits of agreement = − 1.6 to 4.0 dB). SVOP sensitivity had good ability to differentiate healthy and glaucomatous eyes with a 95% CI for area under the curve (AUC) of 0.84 to 0.96, similar to the performance of SAP sensitivity (95% CI 0.86 to 0.97, P = 0.60). For 80% specificity, SVOP had a 95% CI sensitivity of 75.7% to 94.8% compared to 77.8% to 96.0% for SAP. SVOP took considerably longer to perform (514 ± 54 s compared to 267 ± 76 s for SAP). Eye tracking perimetry may be useful for detection of glaucoma but further studies are needed to evaluate SVOP within its intended sphere of use, using an appropriate design and independent reference standard.

## Introduction

Standard Automated Perimetry (SAP) has become the prevailing modality for assessing visual function in glaucoma. The test uses a white static stimulus displayed against a white background, with the contrast of the stimulus varied according to a staircase strategy to determine Differential Light Sensitivity (DLS). SAP has become the ‘gold standard’ perimetric test, yet patients often find it difficult to perform. Although patients accept that visual field testing is important, they find it more demanding than other common glaucoma tests and a qualitative investigation discovered a perception among patients that multiple tests were needed to become comfortable and to gain an accurate representation of their vision^1,2^.

SAP is also subject to considerable test–retest variability with the result that patients may need multiple tests to confidently identify change. Although research on the optimal frequency of clinical tests is limited, it has been suggested that to detect patients progressing at a rapid rate (≥ − 2 dB per year), 3 visual field tests should be conducted per year for the first 2 years of follow up, with more frequent testing required to detect slower rates of change^3^. In reality, visual field testing is often performed less frequently, with a multicentre review of glaucoma clinics in England showing most patients have only one visual field test per year^4^. Similar findings were reported in the US, where even in an academic medical centre, patients had an average of only 1.24 visual tests per year^5^. Despite the guidance highlighting the importance of perimetry, there is evidence that the frequency of visual field testing has decreased, perhaps due to overreliance on imaging. A review of over 150,000 patients with glaucoma, found the odds of having an automated visual field test decreased by 44% from 2001 to 2009, whilst over the same time period there was a 147% increased odds of undergoing computer-based optic disc imaging^6^. Although many factors, particularly organisational and resource constraints, may affect the frequency of visual field testing, the perception that SAP is difficult to perform and unpopular with patients, may contribute to the discrepancy between recommended and observed frequencies of testing.

Over the last decade several groups have explored the possibility of performing perimetry by tracking eye movements^7–13^. Eye trackers have long been used to monitor fixation during visual field assessment, but eye tracking may also be useful for determining whether a stimulus has been seen. We have previously developed a suprathreshold eye tracking perimeter for use in children known as saccadic vector optokinetic perimetry (SVOP)^11^. SVOP was inspired by the work of Damato and colleagues, who in 1989 described a method of computerized perimetry using a moving fixation target presented on a computer monitor^14,15^. Patients were required to position a cursor over a fixation target using a mouse, and stimuli were presented when the cursor was in the correct position. By moving the fixation target, and by using the preceding stimulus as a fixation spot for the next stimulus, a large area of visual field could be tested using a standard computer monitor. A similar approach is used by the more recent Melbourne Rapid Fields test, an FDA approved application that allows testing of 30° of field using the 9.7-inch screen of a tablet computer^16^.

Damato’s perimeter and the Melbourne Rapid Fields require responses to stimuli to be registered by pressing a response button, similar to SAP; however, eye tracking could be used to determine if a stimulus is seen, by detecting eye movements towards the stimulus that occur within a prespecified time of stimuli presentation^11,14–20^. Eye trackers can also determine the position of the eyes relative to the screen to automatically adjust the size and position of stimuli allowing the patient to move their head during testing. Therefore, eye tracking perimetry may provide a more comfortable experience for patients that is likely to be particularly beneficial for those that struggle to maintain fixation for a prolonged period or have difficulty pressing a button to register a response.

The suprathreshold version of SVOP for use in children showed mixed results^11,21,22^. Tailor et al. reported many children were unable to complete testing and agreement with confrontational and Goldmann perimetry was moderate to poor^21^. In contrast, Simkin and colleagues reported a higher proportion of children were able to successfully complete SVOP compared to Goldmann perimetry, with SVOP significantly faster^22^. The differences in results may be due to the inherent difficulty of assessing visual fields in children or due to differences in characteristics of those included in the studies. As adults also report difficulty performing conventional perimetry, we have modified SVOP to develop a threshold version of the test. An advantage of evaluating the test in adults is it is easier to establish a reliable comparison to SAP, and in a previous investigation we found good correlation between threshold sensitivity values obtained with SVOP and SAP and demonstrated SVOP to have good repeatability^19^. Most patients found SVOP comfortable and almost three quarter preferred SVOP compared to standard testing.

The aim of this study was to explore the feasibility of using SVOP to differentiate eyes with glaucoma from healthy controls and compare performance to SAP. Like many evaluations of diagnostic devices, the study was limited by a case–control design and so results may not be generalisable to other settings or groups, and it would be premature to recommend use of eye tracking perimeters in clinical practice^23^; however, the study provides useful data supporting further larger scale evaluation of SVOP.

## Materials and methods

This was a prospective case–control study including patients with glaucoma and healthy volunteers. A two-gate design was used, with patients with glaucoma recruited from the glaucoma clinic at the Princess Alexandra Eye Pavilion, Edinburgh, UK, and healthy participants recruited using the Scottish Health Research Register (SHARE), a national register of volunteers interested in participating in research. Patients attending the glaucoma clinic were invited to participate by their treating clinician. All participants provided written informed consent prior to enrolment and all study methods were prospectively approved by the South-East Scotland Research Ethics Committee (reference 13/SS/0045). The study adhered to the tenets of the Declaration of Helsinki.

Patients attending the glaucoma clinic underwent a comprehensive ophthalmic examination, including best-corrected visual acuity, slit lamp biomicroscopy, intraocular pressure (IOP) measurement using Goldmann applanation tonometry, gonioscopy and dilated fundoscopy. The reference standard was a diagnosis of glaucoma made by the treating glaucoma consultant based on the comprehensive ophthalmic examination and all patients with glaucoma were required to have a glaucomatous visual field defect on SAP using the Humphrey Field Analyzer (HFA) SITA Fast 24-2 test (Carl Zeiss Meditec, Inc., Dublin, CA). The treating glaucoma consultant did not have access to the SVOP test results. Patients with non-glaucomatous ocular or non-ocular conditions, such as neurological disease, that might affect the visual field were excluded. Healthy participants were required to have no previous history of significant eye disease, no known history of visual field defect and no neurological conditions that might affect the visual field.

All participants had SAP using a Humphrey Field Analyzer (HFA) 750i (*Carl Zeiss Meditec, Dublin, CA*) with the 24-2 test pattern and the SITA Fast algorithm. The index test, SVOP, was performed at the same visit using a threshold SVOP research device, described in detail in previous publications^19^. All patients completed SAP and SVOP in both eyes, with the order of testing randomised. A random group of participants then performed repeat SAP and SVOP in one eye, randomly chosen for healthy subjects and with the worse affected eye selected in those with glaucoma. Visual field tests were reviewed for reliability and artefacts. SAP tests with ≥ 15% false positives or ≥ 20% fixation losses were considered unreliable and excluded. False negative rates were not considered due to evidence indicating false negative rates are more strongly indicative of glaucoma severity than attentiveness^24^. SVOP does not provide information about false positives or fixation losses as it does not require the patient to maintain fixation on a single point or register a response with a button. Inherent to the test, SVOP accounts for fixation by not presenting a further stimulus until fixation is achieved.

### Saccadic vector optokinetic perimetry (SVOP)

The threshold SVOP device consists of a personal computer with a 24″ high-resolution Liquid Crystal Display (LCD) screen (Eizo ColorEdge CG243W, Hakusan, Japan) and a 60 Hz eye tracker (X2-60 model, Tobii Technology, Stockholm, Sweden)^19,20^. A 60 Hz eye tracker has a sampling rate of 1 data point every 16 ms.

**Figure 1:**
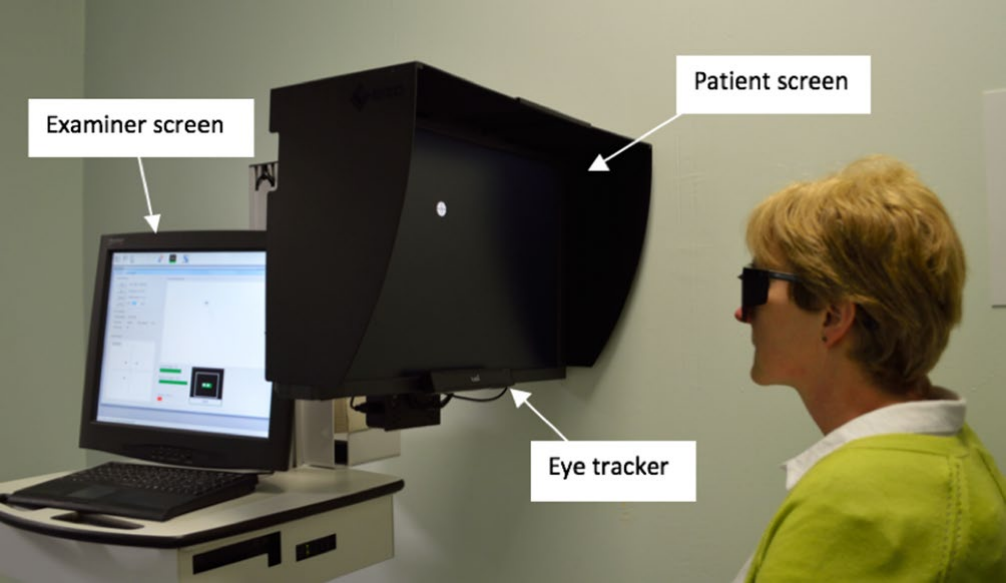
The threshold saccadic vector optokinetic perimetry (SVOP) instrument showing the patient screen, eye tracker position and examiner screen.

Participants were seated in front of an LCD screen with their eyes aligned with the screen’s centre at an initial distance of 55 cm (Fig. 1). Written informed consent was obtained for publication of this identifying image in an open-access publication. Each eye was tested separately using custom made spectacles, which occluded the non-test eye with a darkened infrared bandpass filter. This filter enabled the eye tracker to detect the position of the occluded eye, while blocking the occluded eye from seeing the stimuli. Before testing commenced there was a 20 s demonstration followed by an eye-tracker calibration sequence. Calibration enabled the geometric characteristics of eyes to be estimated for accurate gaze point calculation. During calibration the user was asked to look at specific points on the screen, with the sequence taking approximately 30 s. The calibration sequence was included in the reported SVOP test time.

During testing participants were instructed to look towards any peripheral stimulus seen. The eye tracker evaluated gaze responses to the stimuli and software determined whether the stimulus had been seen based on the direction and amplitude of the gaze response. Whether or not the stimulus was seen was determined based on the direction and amplitude of the change in eye position relative to the stimulus and the point of fixation. The start of a fixation change was defined as the start point of a greater than 50 pixels change in gaze and the end location was defined by the point at which 5 consecutive gaze data samples were separated by a distanced of < 50 pixels after the detection of a fixation change. Stimuli were equivalent to Goldmann size III and each stimulus was presented for 200 ms using coordinates equivalent to the SAP 24–2 test pattern. As the eye tracker provides data on eye location, the size and position of stimuli were able to be automatically adjusted to compensate for changes head position during testing. A stimulus duration of 200 ms was selected as it is the same duration as HFA and previous work has shown visual processing speed and speed of the saccade response is sufficiently fast to reach a stimulus within this time period^25,26^. Saccades larger than 5° take only approximately 20 to 30 ms, with an additional 2 ms for each additional degree.

Grey-scale level colours were produced by the LCD screen by setting red, green and blue (RGB) levels to equal each other and luminance was varied by adjusting RGB levels. The maximum level of luminance was obtained by setting the RGB level to 255, 255, 255 and the minimum to 0, 0, 0. Screen calibration was performed using a Look-Up Table to pair grey levels of each pixel to the corresponding required background (10 Cd/m^2^) and stimulus luminance levels. To minimize the risk of variability in luminance affecting results, uniformity of the display screen was assessed using a luminance meter (L203 photometer, Macam Photometrics Ltd, Livingston, UK). Luminance values were examined for different grey-scale levels to ensure LCD RGB values corresponded to stimulus luminance used by SAP. For example, a stimulus of 20 dB (41.9 Cd/m^2^) was found to be equivalent to an LCD RGB value of 136, 136, 136. Stimuli luminance replicated the luminance values corresponding to 14 to 40 dB with SAP, with the background to stimuli luminance ratio also replicated. The LCD display was unable to display stimuli with luminance < 14 dB due to the maximum intensity being limited by the maximum luminance of the LCD backlight.

Thresholds were obtained using a 4–2 bracketing strategy and began by testing four seed locations (one in each quadrant), which were then used to set the starting stimulus luminance levels for neighbouring locations which in-turn were used to calculate the remaining starting luminance levels. The SVOP stimulus intensity and background intensity values were matched in luminance to those of SAP to allow direct comparison. Version 2.0 of the SVOP threshold software was used for all participants.

### Data analysis

The relationship between SAP and SVOP visual field sensitivity was examined using scatter plots and pointwise linear regression. Histograms were also constructed to survey the distribution of differences in sensitivity between SAP and SVOP for each visual field test location. All results were transposed to right eye format. Bland–Altman plots were used to compare results from SAP and SVOP and determine 95% limits of agreement.

Receiver Operating Characteristic (ROC) curves were constructed to assess the ability of SVOP and SAP to differentiate participants with glaucoma from healthy controls^27^. A ROC curve is a plot of sensitivity versus 1-specificity across threshold values, showing the intrinsic capacity of the test to discriminate diseased and nondiseased status^28^. The area under the ROC curve (AUC) was used to summarise the diagnostic accuracy. To account for differences in age between participants with glaucoma and controls, the covariate effect of age was adjusted for using the ROC generalised linear regression method described by Pepe et al.^27,28^ and first described for evaluation of glaucoma diagnostic tests by Medeiros et al.^29^ Results were reported with age set at the sample mean.

ROC regression used a 1000 repetition bootstrap technique to estimate 95% confidence intervals. As measurements from both eyes of the same subject are likely to be correlated, the cluster of data for each participants was used as the unit of resampling when calculating confidence intervals^29^. All statistical analyses were performed with commercially available software (STATA version 12; StataCorp LP, College Station, TX). The α level (type I error) was set at 0.05.

## Results

61 eyes of 31 participants with glaucoma and 47 eyes of 24 healthy participants were included in the study. 26 of 55 participants (47.3%) were female. There was no difference in gender between healthy and glaucomatous participants, however those with glaucoma were significantly older (P < 0.001). Demographic and clinical details of all participants are shown in Table 1.

**Table 1:** Demographic and clinical details of all participants included in the study.

	Normal (n = 47 eyes, 24 subjects)	Glaucoma (n = 61 eyes, 31 subjects)	P-value
Age (years)	66.0 ± 5.6	72.3 ± 7.9	< 0.001
Gender (female, n)	9 (37.5%)	17 (54.8%)	0.122
SAP MD (dB)	0.0 ± 0.8	− 8.7 ± 7.4	< 0.001
Mean SAP sensitivity (dB)	29.6 ± 0.9	23.3 ± 4.4	< 0.001
Mean SVOP sensitivity (dB)	28.4 ± 1.3	22.1 ± 4.3	< 0.001
SAP test duration (s)	190 ± 34	267 ± 76	< 0.001
SVOP test duration (s)	533 ± 167	514 ± 154	0.545

*SAP* standard automated perimetry, *MD* mean deviation, *dB* decibels, *SVOP* saccadic vector optokinetic perimetry, *s* seconds.

**Figure 2:**
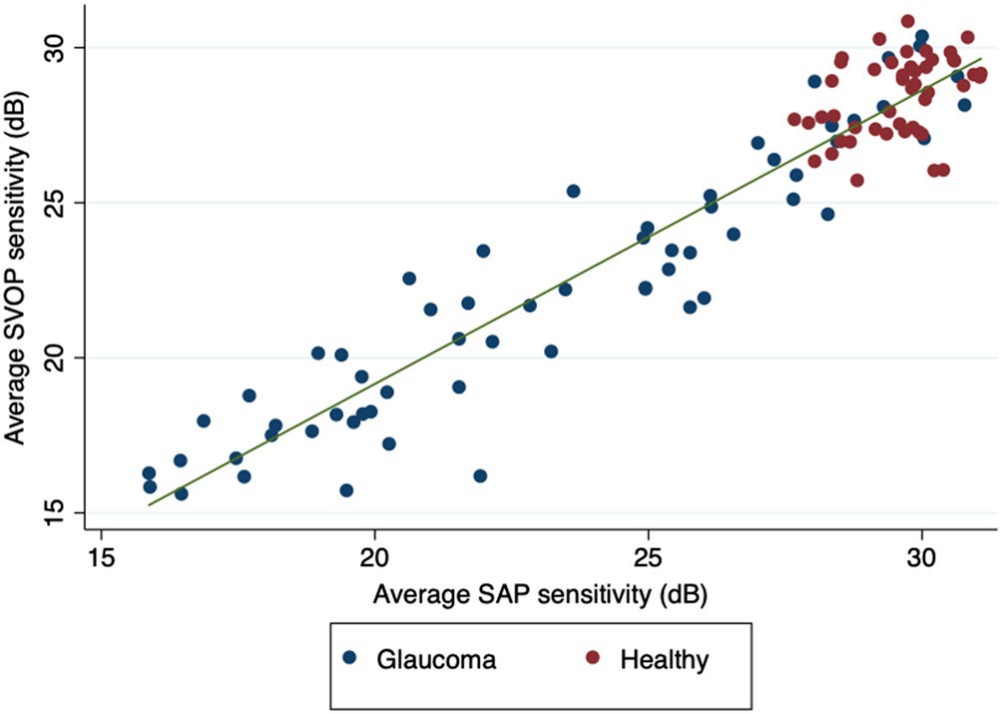
Relationship between average standard automated perimetry (SAP) and saccadic vector optokinetic perimetry (SVOP) sensitivity for healthy and glaucomatous participants.

**Figure 3:**
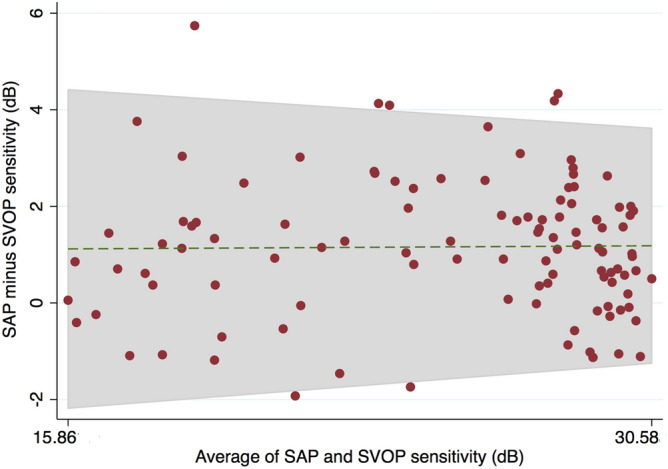
Bland Altman plot for visual field sensitivity comparing standard automated perimetry (SAP) and saccadic vector optokinetic perimetry (SVOP).

Mean SAP mean deviation (MD) in glaucomatous eyes was − 8.7 ± 7.4 dB, with mean SAP and SVOP sensitivities of 23.3 ± 0.9 dB and 22.1 ± 4.3 dB respectively. There was strong correlation between mean SAP and SVOP sensitivities (r = 0.951, P < 0.001) (Fig. 2). On average, SAP sensitivity was 1.2 ± 1.4 dB higher than SVOP sensitivity, with 95% limits of agreement of − 1.6 to 4.0 dB (Fig. 3). Bland–Altman analysis may lead to proportional bias, which is present when the difference in values is related to their average. There was no evidence of proportional bias demonstrated by the lack of a significant relationship between the mean of mean SVOP and SAP sensitivity and the difference between SVOP and SAP sensitivities (R^2^ = 0.0002, P = 0.886)^30^.

SVOP had good repeatability with a concordance correlation coefficient (rho_c) of 0.98 (95% CI 0.97 to 1.00, P < 0.001, Pearson’s r = 0.987) and a mean difference between repeat measures of 0.37 ± 0.91 dB (95% limits of agreement of − 1.41 to 2.16 dB). In comparison, SAP had concordance correlation coefficient of 0.99 (95% CI 0.98 to 1.0, P < 0.001, Pearson’s r = 0.991) and a mean difference between repeat measures of 0.13 ± 0.87 dB (95% limits of agreement of − 1.56 to 1.83 dB).

**Figure 4:**
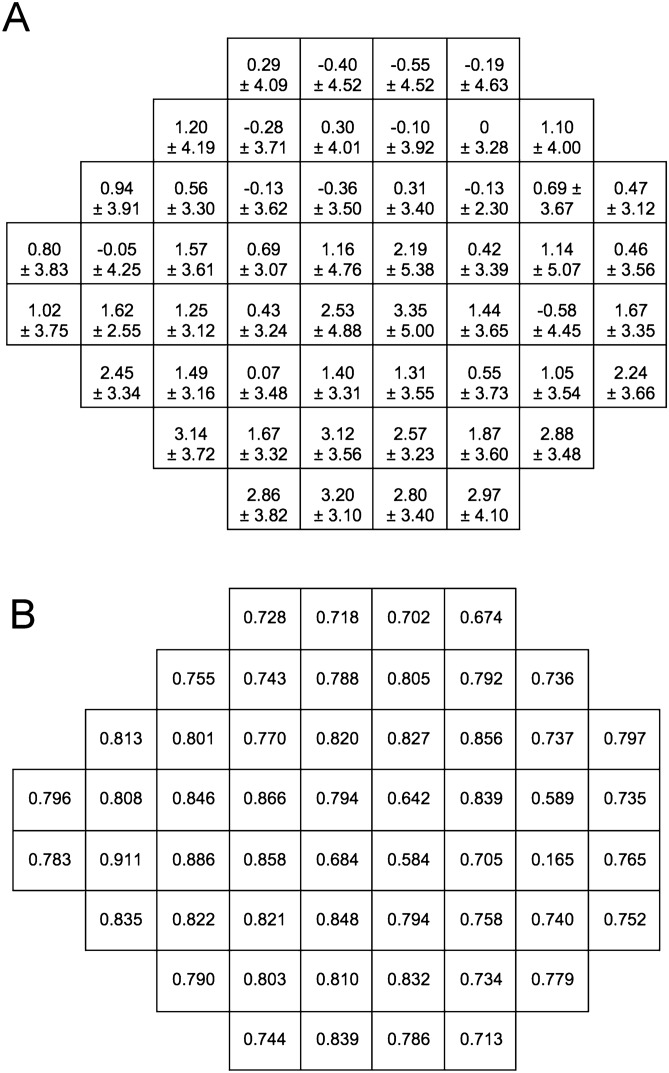
Mean (± standard deviation (SD)) differences (in decibels) between threshold sensitivities from standard automated perimetry (SAP) and saccadic vector optokinetic perimetry (SVOP) at each location of the visual field (**A**) and correlation between SVOP and SAP sensitivity for each test location (**B**). Negative values indicate threshold sensitivity measured by SVOP was lower than that measured by SAP.

**Figure 5:**
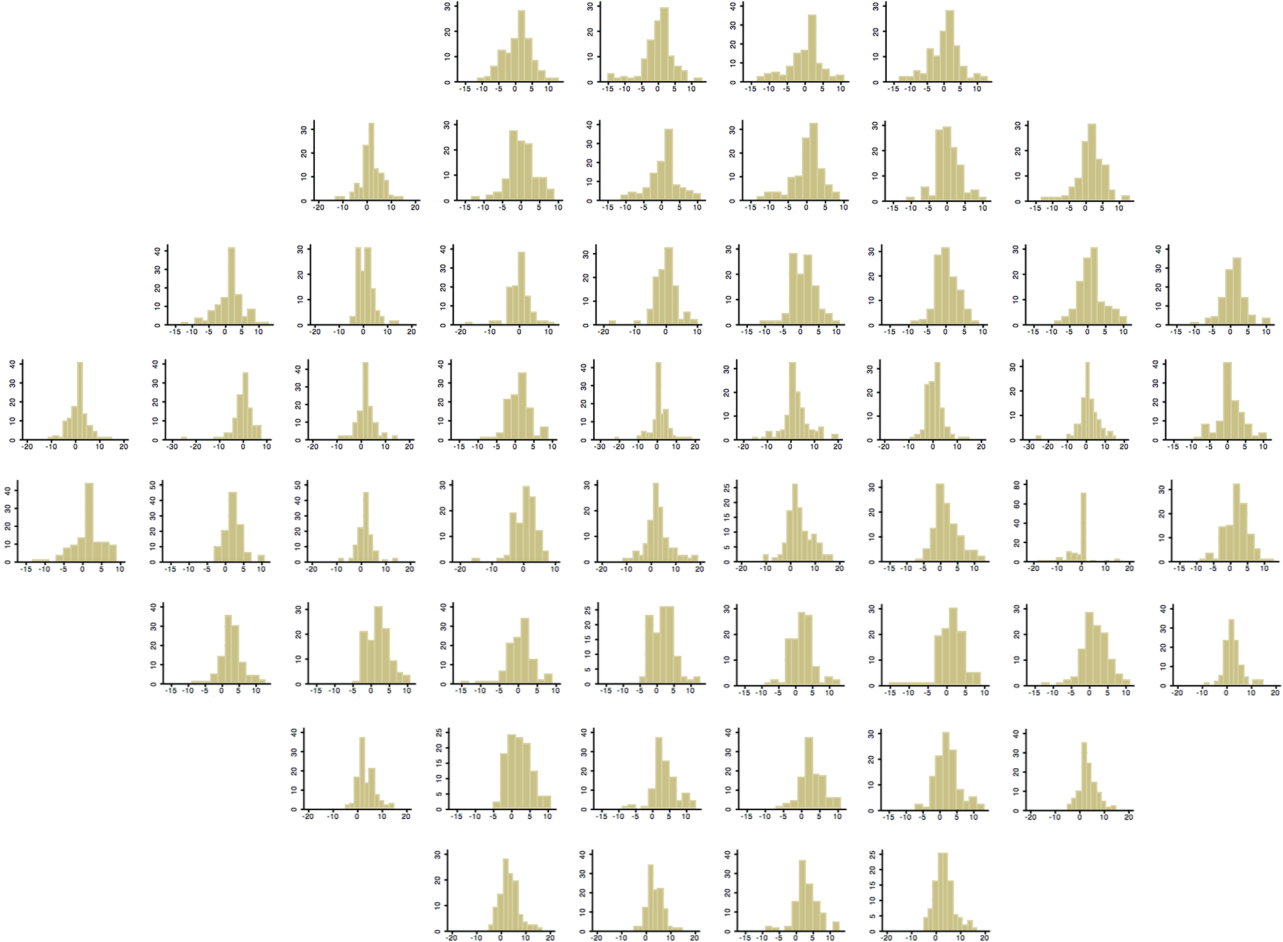
Histograms showing the distribution of point by point differences in sensitivity (in decibels) between standard automated perimetry (SAP) and saccadic vector optokinetic perimetry (SVOP) for all participants and for all test locations. The difference in sensitivity between SVOP and SAP is shown on the x-axis and the frequency of differences on the y-axis.

Pointwise analysis showed the average (± SD) difference in SAP and SVOP sensitivities ranged from a low of 0 ± 3.28 dB to a high of 3.35 ± 5.00 dB (Fig. 4A), with correlation between tests ranging from 0.58 to 0.91, excluding the blind spot (Fig. 4B). Excluding the blind spot, all but 5 test locations had a correlation of greater than 0.7. Histograms showing the distribution of differences in sensitivity between SVOP and SAP for each test location are shown in Fig. 5. Agreement between tests was worse in the four most central test locations than at other locations. The mean differences in sensitivity were 1.16 ± 4.76 dB, 2.19 ± 5.38 dB, 2.53 ± 4.88 dB and 3.35 ± 5.00 dB for the central 4 test locations compared to a mean difference of 0 ± 3.28 dB in the test location with best agreement (Fig. 4A).

**Figure 6:**
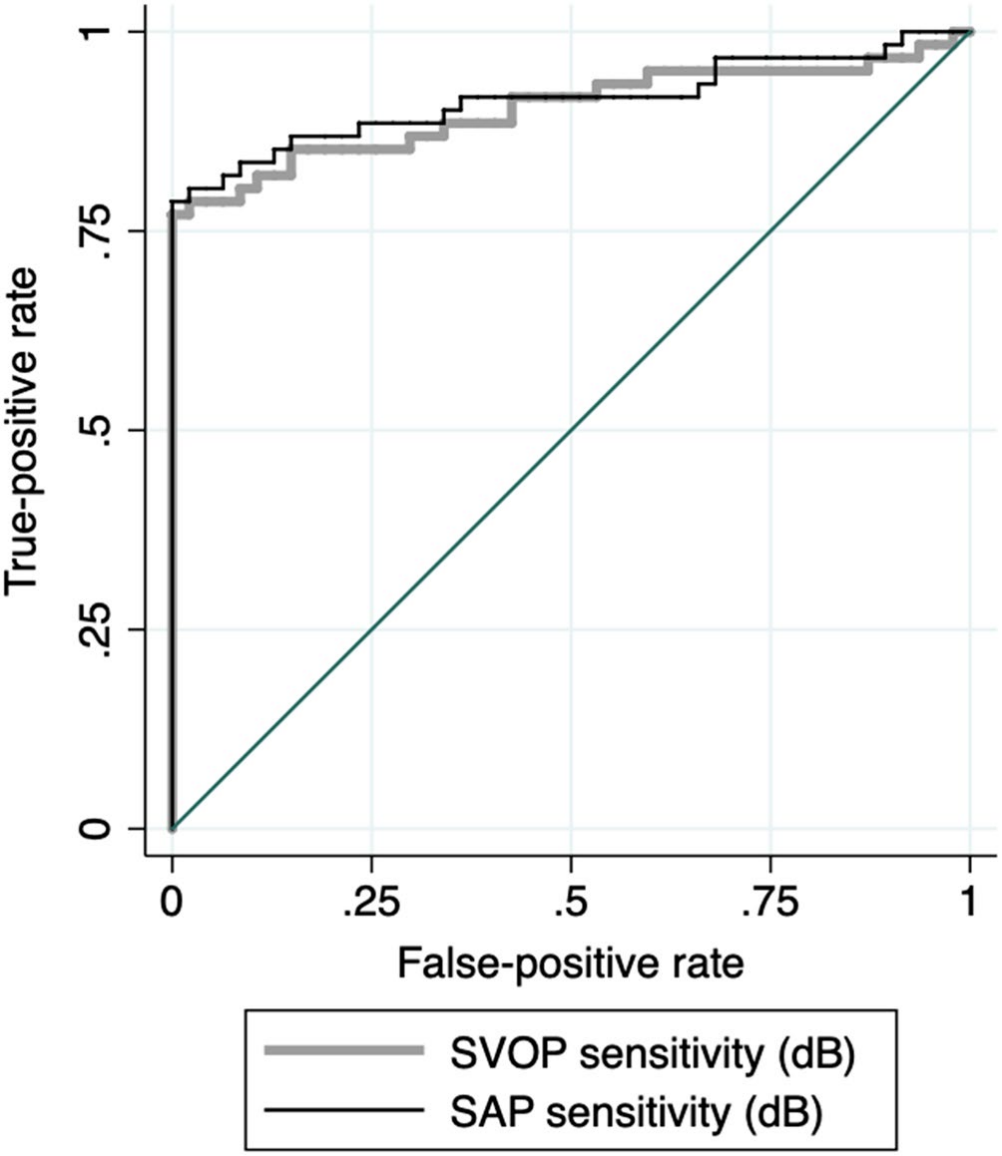
Receiver operating characteristic curve comparing the ability of standard automated perimetry (SAP) and saccadic vector optokinetic perimetry (SVOP) average sensitivities to differentiate eyes with and without glaucoma (area under the receiver operating characteristic curve (AUC) = 0.90, 95% CI 0.84 to 0.96 for SVOP versus 0.92, 95% CI 0.86 to 0.97 for SAP, P = 0.602).

SVOP mean sensitivity had good ability to differentiate healthy and glaucomatous eyes included in the study with an AUC of 0.90 (95% CI 0.84 to 0.96), which was not significantly different to the performance of SAP mean sensitivity (AUC = 0.92, 95% CI 0.0.86 to 0.97, P = 0.602) (Fig. 6). SVOP had 85.2% (95% CI 75.7% to 94.8%) sensitivity for 80% specificity, compared to 86.9% sensitivity (95% CI 77.8% to 96.0%) for 80% specificity for SAP. For 90% specificities, sensitivities were 80.3% (95% CI 69.2% to 91.4%) and 83.6% (95% CI 73.4% to 93.8%) for SVOP and SAP respectively.

Despite its good performance, SVOP tests took signficantly more time to complete than SAP with average test times of 522 ± 159 s and 233 ± 72 s (P < 0.001) respectively. There was no significant relationship between age and test time for SVOP (R^2^ = 0.003, P = 0.554) but older participants were slower at SAP (R^2^ = 0.271, P < 0.001). Each decade of increasing age was associated with 49 s slower SAP test time (95% CI 24 to 65 s, P < 0.001). SAP also took longer in those with worse disease severity, on average taking 12 s longer for every 1 dB worse average SAP sensitivity (95% CI 14 to 10 s, P < 0.001). In multivariable analysis, disease severity remained signficantly associated with SAP test duration (P < 0.001), whereas age did not (P = 0.092). The duration of SVOP was not influenced by average sensitivity (P = 0.697) meaning the difference in test times between SAP and SVOP was greater in healthy participants compared to those with glaucoma (Table 1).

## Discussion

The results of this study suggest that eye tracking perimetry could have a future role in aiding the detection of glaucoma, though limitations of the study design, particularly the small sample size and case–control design, mean there remains insufficient evidence for use in current clinical practice. Overall, threshold sensitivities obtained from SVOP and SAP showed good agreement, and both SVOP and SAP had similar ability to differentiate healthy and glaucomatous eyes, despite inclusion of SAP in the diagnostic reference standard. SVOP mean sensitivity achieved an AUC of 0.90 compared to 0.92 for SAP, with 85.2% and 86.9% sensitivities for 80% specificity for SVOP and SAP respectively, though due to the small sample size confidence intervals were wide.

Were it possible to replicate this performance in clinical practice, SVOP would likely be a useful diagnostic aid, however, it is important to acknowledge that case–control studies tend to overestimate performance, particularly when including patients with advanced disease, and when excluding patients with certain characteristics, some of which may be risk factors for the disease, e.g. high myopia. The average SAP MD of patients included in this study was − 8.72 dB, however, the study included patients with a wide range of visual field loss (Fig. 2). Due to the limited sample size we were unable to explore the performance of SVOP across the range of disease severities and this would be an important area for future study given that performance is likely to be reduced in early disease. Overall, the performance of SVOP and SAP may be similar but if SVOP has worse performance in detecting early disease, it may be of less value.

The diagnostic accuracy of a test is not a fixed property and will vary depending on setting, prior testing and characteristics of participants^23^. These are important considerations when deciding whether to adopt a test in a specific environment. As a case control study is an artificial construct, it does not replicate the use of a test in the setting of proposed used, and therefore does not provide sufficient evidence to justify use of SVOP in clinical practice. Nevertheless, the results, coupled with previous studies showing a patient preference for SVOP^20^, suggest that larger scale studies of use of eye tracking perimeters in a clinical pathway are warranted.

The performance of a test is highly influenced by choice of reference standard and the ideal reference standard would be independent of perimetry, for example, classification on optical coherence tomography, or progressive changes on masked observation of optic disc stereophotographs. The chosen reference standard for the current study was a diagnosis of glaucoma made by the treating glaucoma consultant based on a comprehensive ophthalmic examination, with all patients required to have a glaucomatous visual field defect on SAP. The requirement for a defect on SAP would introduce positive bias in assessment of the ability of SAP mean sensitivity to differentiate glaucomatous and healthy eyes and would also introduce bias in favour of any perimetric device in which results correlated with SAP. If a structural marker of glaucoma had been selected as the reference standard, the performance of SAP and SVOP would likely have been reduced. However, in this pilot study, it was deemed most important to determine whether SVOP was able to identify patients with glaucoma with a visual field defect on SAP, and to determine the number of false positives. It is patients with a defect on SAP that are at most risk of visual impairment from glaucoma, though we acknowledge that the choice of reference standard meant we were unable to determine whether SVOP might have better ability to detect glaucoma than SAP or examine the potential for SVOP to detect glaucoma before it is evident on conventional perimetry (pre-perimetric glaucoma).

McTrusty and colleagues previously evaluated the ability of threshold SVOP to identify normal versus abnormal SAP, reporting a sensitivity of 98% and specificity of 78%^20^. The lower sensitivity in the present study likely reflects the difference in reference standard, with McTrusty and colleagues evaluating the ability to detect and abnormal visual field rather than the ability to differentiate glaucomatous and healthy individuals. In addition, McTrusty and colleagues defined an abnormal SVOP based on masked grading rather than the summary index of average sensitivity and did not perform ROC analysis.

The good performance of SVOP likely reflects a high level of agreement with SAP. On average, DLS measured by SAP was 1.16 dB higher than DLS measured by SVOP, with 95% limits of agreement of − 1.64 to 3.96 dB (Fig. 3). Pointwise analysis revealed similarly good agreement between SVOP and SAP as the analysis of mean sensitivity, however, from Fig. 4 it is apparant that agreement was worse for the four central test loctations, which are arguably the most important for visual function. Figure 4A shows that SAP sensitivity was on average 3.35 dB higher than SVOP in the inferotemporal central test location, with a corresponing correlation of only 0.58. In contrast, other test points had average differences of 0 dB between SAP and SVOP and correlation as high as 0.91. Due to the lack of an independent measure of DLS it is not possible to be certain which of SAP or SVOP was closest to actual DLS, however, we observed that the eye tracker used with the SVOP device sometimes failed to detect small amplitude eye movements in the central field. The eye gaze error reported by the eye tracker manufacturer is 0.58 degrees, however error may be greater in more uncontrolled conditions, such as during an SVOP test where a patient’s head may move. The result is that some stimuli that were perceived by the subject may not have been recorded as ‘‘seen’’ by SVOP. The eye tracker used in this study was only 60 Hz and it is likely that agreement between SVOP and SAP in the central field locations could be improved by improvements in eye tracking technology but this would need formal evaluation.

A further limitation of SVOP was the longer test time compared to the SAP SITA Fast strategy. SVOP took over twice as long to perform as SAP, with an average test time per eye of 8.7 min for SVOP compared to 3.9 min for SAP. In addition, SVOP test times were similar in patients with glaucoma and healthy individuals, whereas SAP was faster in healthy subjects. Were SVOP to be used in a screening setting where a large number of healthy subjects would be tested, this would have significant implications for total test times and would likely make the current SVOP test strategy impractical outside of a research setting. The difference in test times is a consequence of the exhaustive staircase search strategy employed by SVOP rather than the optimised threshold estimate provide by SITA Fast and similar strategies could potentially be adopted to improve the SVOP test duration. It is interesting that despite the significantly longer test time, a previous study reported 71% of patients preferred SVOP to SAP^20^.

A further potential limitation of eye tracking perimetry is the sampling error inherent in eye trackers, which may mean that small eye movements go undetected. A 60 Hz eye tracker has a sampling rate of 1 data point every 16 ms, meaning that fast saccades, which take only 20 to 30 ms, may not have been detected. Use of an eye tracker with a higher temporal frequency would reduce sampling error and may improve accuracy; however, an eye tracking perimeter may not need to detect every small saccade, as long as it can determine whether a stimulus has been perceived and that it has been perceived while the patient is fixing on a fixation spot. Stimuli were presented for 200 ms giving a potential of 200/16 = 12.5 data points during each stimulus presentation. 5 consecutive data samples (80 ms of sampling) separated by < 50 pixels were required to define an endpoint of change in gaze, meaning the maximum time available for an eye movement between first appearance of a stimulus and fixation on the stimulus was only 200–80 = 120 ms. This was deemed sufficient time for a saccade to each the stimulus but insufficient for smooth pursuit.

Using an ealier suprathreshold version of SVOP, Tailor and colleagues highlighted that patients with abnormal saccades may have artefactual non-specific visual field defects^9^. Performing any form of automated perimetry in patients with abnormal eye movements is lilkely to be challenging, however, eye tracking perimetry may be particularly unrelaible in such individuals. As no participants in the current study had eye movement disorders we were not able to evaluate their effect on SVOP relaiblity but this is likely to be a limitation of the test.

In summary, this study provides evidence that eye tracking perimetry using SVOP may be useful for aiding glaucoma detection, with performance in this case control study, similar to conventional automated perimetry. The eye tracking perimeter has the advantage of not requiring the patient to place their chin on a rest or press a button when a stimulus is seen and in previous studies has been found to be preferred by patients, however, a major limitation was that SVOP took considerably longer to perform. The study was limited by a case–control design, small sample size, and imperfect reference standard, but nevertheless the results show that eye tracking perimetry is feasbile and provide a foundation for potential further studies examining performance of eye tracking perimetry in a clinical pathway.

## Data Availability

The datasets generated during and/or analysed during the current study are available from the corresponding author on reasonable request.
